# *MICAL2* is a novel human cancer gene controlling mesenchymal to epithelial transition involved in cancer growth and invasion

**DOI:** 10.18632/oncotarget.6577

**Published:** 2015-12-12

**Authors:** Sara Mariotti, Ivana Barravecchia, Carla Vindigni, Angela Pucci, Michele Balsamo, Rosaliana Libro, Vera Senchenko, Alexey Dmitriev, Emanuela Jacchetti, Marco Cecchini, Franco Roviello, Michele Lai, Vania Broccoli, Massimiliano Andreazzoli, Chiara M. Mazzanti, Debora Angeloni

**Affiliations:** ^1^ Institute of Life Sciences, Scuola Superiore Sant'Anna, 56124 Pisa, Italy; ^2^ U.O.C. Anatomia Patologica, Azienda Ospedaliera Universitaria Senese, Policlinico Le Scotte, 53100 Siena, Italy; ^3^ U.O.C. Anatomia Patologica, Azienda Ospedaliera Universitaria Pisana, 56100 Pisa, Italy; ^4^ BIOS Doctoral School in Life Sciences, University of Pisa, 56124 Pisa, Italy; ^5^ Engelhardt Institute of Molecular Biology, Russian Academy of Sciences, 119991 Moscow, Russia; ^6^ NEST, National Enterprise for nanoScience and nanoTechnology, CNR and Scuola Normale Superiore, 56127 Pisa, Italy; ^7^ Department of Human Pathology and Oncology, University of Siena, 53100 Siena, Italy; ^8^ Pisa Science Foundation, 56100 Pisa, Italy; ^9^ DIBIT H San Raffaele, 20132 Milan, Italy; ^10^ Department of Biology, University of Pisa, 56127 Pisa, Italy

**Keywords:** MICAL2, kidney cancer, gastric cancer, epythelial to mesenchymal transition, metastasis

## Abstract

The MICAL (Molecules Interacting with CasL) proteins catalyze actin oxidation-reduction reactions destabilizing F-actin in cytoskeletal dynamics.

Here we show for the first time that *MICAL2* mRNA is significantly over-expressed in aggressive, poorly differentiated/undifferentiated, primary human epithelial cancers (gastric and renal). Immunohistochemistry showed MICAL2-positive cells on the cancer invasive front and in metastasizing cancer cells inside emboli, but not at sites of metastasis, suggesting *MICAL2* expression was 'on' in a subpopulation of primary cancer cells seemingly detaching from the tissue of origin, enter emboli and travel to distant sites, and was turned 'off' upon homing at metastatic sites.

*In vitro*, *MICAL2* knock-down resulted in mesenchymal to epithelial transition, reduction of viability, and loss of motility and invasion properties of human cancer cells. Moreover, expression of MICAL2 cDNA in MICAL2-depleted cells induced epithelial to mesenchymal transition.

Altogether our data indicate that *MICAL2* over-expression is associated with cancer progression and metastatic disease. MICAL2 might be an important regulator of epithelial to mesenchymal transition and therefore a promising target for anti-metastatic therapy.

## INTRODUCTION

The cure of most cancers will ultimately depend on the capability to eradicate disseminated secondary tumors that do not respond to therapy. Our current inability to do so motivates the search for target genes implicated in cancer progression.

Cytoskeleton remodeling is a crucial event in metastasis progression because it is instrumental in regulating fundamental cell properties, from proliferation and/or differentiation to cell-cell and cell-substrate adhesion, motility and invasion. Tight regulation of actin dynamics relies on the activity of different actin-binding proteins controlling the transition from globular (G) to filamentous (F) actin, as well as nucleation, capping, severing, elongation and crosslinking of actin filaments [1, 2].

Recently, the family of Molecules Interacting with CasL (MICAL) [3, 4], was implicated in the regulation of actin cytoskeleton dynamics [5, 6]. MICALs have a unique structure that combines an N-terminal flavoprotein mono-oxygenase (MO) domain [7, 8] with other protein-protein interaction modules that network with cytoskeletal and signaling partners [3, 4]. *Drosophila* MICAL (D-MICAL) exerts oxidation-reduction (Redox) reactions to directly oxidize two methionine residues of actin, thereby destabilizing F-actin and inhibiting local assembly. D-MICAL activity is necessary for spatial guidance of the axonal growth cone, a highly motile sensory structure localized at the axon tip, essential for guiding neurons to their synaptic targets [9]. We reasoned that the striking capability of MICAL to directly and mechanistically connect oxygen availability with F-actin depolimerization and hence cytoskeleton dynamics might be extremely important also for metastatic cancer cells whose motility is increased as part of epithelial to mesenchymal transition (EMT). In fact, during the growth of solid tumors challenging micro-environmental factors (hypoxia, acidity, inflammatory cytokines, etc) stimulate cancer cells to enact escape adaptive strategies. Lead by a regulated genetic/epigenetic program, epithelial cells loose epithelial markers, cell-cell and cell-extracellular matrix (ECM) interactions, undergo cytoskeleton reorganization, gain gene expression profile, morphological and functional characteristics of mesenchymal cells, and leave the primary tumor site [10]. Both EMT and its opposite, mesenchymal to epithelial transition (MET), are implicated in developmental and pathological contexts [10]. During MET, mesenchymal markers are down-regulated, cell motility decreases and cells adopt epithelial characteristics [10].

Up to now, MICALs involvement in human cancer was completely unexplored except for a report of *MICAL2* splicing variants identified in prostate cancer [11]. While this work was in submission, it was published that MICAL-LIKE2, a protein of the MICAL family that shares sequence homology with MICAL2 but lacks the aminoterminal mono-oxygenase domain, is over-expressed in ovarian cancer and when silenced induces MET in ovarian cancer cells [12].

Given the relevance of MICAL proteins to cell motility and the complete lack of information in the context of human primary cancer, we were compelled to understand whether MICAL2 activity might affect cancer cell motility and/or invasion activity, two properties crucial for determining the magnitude of cancer clinical effect. So we set out to investigate a possible involvement of MICALs in human epithelial cancer. We started with MICAL2 because of its basal activation not down-regulated by self-inhibitory activity present in MICAL1 and possibly in MICAL3 [13–15]. This feature suggested that deregulated expression might be sufficient to derange MICAL2 function, a trait in common to other actin-binding proteins involved in cancer.

## RESULTS

### *MICAL2* is variably expressed in human normal and cancer tissues

To search for novel genes involved in metastasis, we investigated the possible role of *MICAL2* in cancer.

Interrogating web-based, expression databases we found *MICAL2* mRNA variably and almost ubiquitously expressed in normal tissues, including stomach, lung and kidney (UniGene: http://www.ncbi.nlm.nih.gov/UniGene/ESTProfileViewer.cgi?uglist=Hs.501928), with noticeable expression variations in several types of human cancer (IST Online: http://ist.medisapiens.com/#ENSG00000133816), together with several outliers, also in lung and gastric cancer (GC), indicating possible patient subpopulations within each cancer type. *In vitro*, comparing *MICAL2* mRNA z-score across the NCI-60 panel, we found *MICAL2* variably expressed in different cell lines (CellMiner: http://discover.nci.nih.gov/cellminer/analysis.do). Interestingly, *MICAL2* was down-regulated in epithelial-like breast cancer cells MCF7 and T47D, but up-regulated in breast cancer cells with mesenchymal features MDA-MB 231, BT-549 and HS578T.

We further explored the clinical significance of *MICAL2* in human cancer performing comparative real time PCR (QRT-PCR) in normal/tumor paired biopsies of patients affected with three major types of human cancers: of the lung (non small cell lung cancer, NSCLC: Adenocarcinoma, AC; squamous cell carcinoma, SCC), kidney (clear cell renal cell carcinoma, ccRCC; papillary renal cell carcinoma, pRCC), and stomach (diffuse and intestinal histotypes).

**Figure 1:**
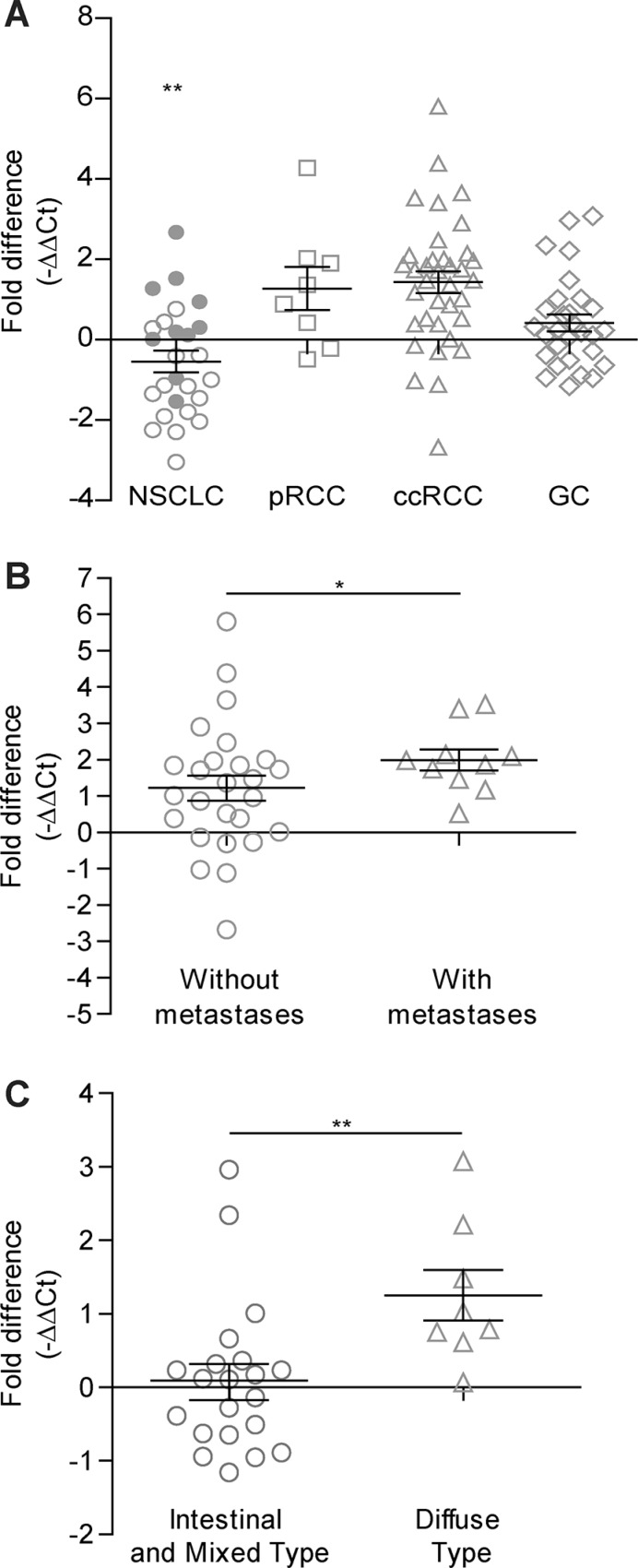
QRT-PCR gene expression analysis of *MICAL2* in lung, kidney and stomach cancers (**A**) *MICAL2* deregulated expression in NSCLC (circles, *N* = 26), pRCC (squares, *N* = 8), ccRCC (triangles, *N* = 36) and GC (diamonds, *N* = 29). In NSCLC patients (10 AC and 16 SCC), *MICAL2* is under-expressed in SCC (open circles, *N* = 16) in comparison to AC (thick circles, *N* = 10) patients. (**B**) *MICAL2* mRNA level in ccRCC (*N* = 36) samples, against normal mucosa, was higher in patients with metastasis (triangles) in contrast to those without (circles). (**C**) In GC (*N* = 29), *MICAL2* was over-expressed in Diffuse type (triangles) versus Intestinal or Mixed type (circles) samples. In all graphs, horizontal lines denote mean and SEM. *p ≤ 0.05, **p ≤ 0.01, ***p ≤ 0.001 (Mann-Whitney non-parametric test).

In 27 NSCLC patients (11 AC, 16 SCC), we found statistically significant under-expression of *MICAL2* in SCC primary tumors compared with AC (*p* < 0.01, Figure 1A). We wondered whether it could be associated with SCC lesser tendency to be invasive compared with AC, but this was not investigated further.

*MICAL2* was moderately to highly over-expressed in 6/8 pRCC patients (75%, Figure 1A). In 36 ccRCC primary samples, we found *MICAL2* significantly over-expressed in patients ‘with metastases’ versus ‘without metastases’ (*p* < 0.05, Figure 1B).

Three histotypes of GC are described [16]: 1) intestinal, (cancer cells are slow-growing, well differentiated, forming tubular or papillary gland structures), 2) diffuse (cancer cells are more aggressive, poorly differentiated, with a tendency to spread); 3) mixed (with growing characteristics common to both diffuse and intestinal histotypes). In 30 GC samples, *MICAL2* was found over-expressed in the diffuse histotype compared with intestinal and mixed histotypes together (*p* < 0.01, Figure 1C). Further, *MICAL2* was found over-expressed (*p* < 0.05) in samples of poorly differentiated and undifferentiated subtype G3 and G4 grade score (WHO criteria, [17]) as opposed to well and moderately differentiated G1 and G2 score [17], not shown.

Overall, QRT-PCR showed *MICAL2* up-regulation in poorly differentiated, aggressive tumor of the lung, kidney and stomach compared with paired normal tissues, and in association with metastasis.

### MICAL2 is expressed at the cancer invasive front, and inversely correlates with expression of proliferation marker Ki-67

To identify the cellular distribution of MICAL2 expression, we performed immunohistochemistry (IHC) analyses of histological sections of gastric and kidney cancer, using specific anti-MICAL2 polyclonal antibodies generated in our laboratory (Supplementary Figure 1).

**Figure 2:**
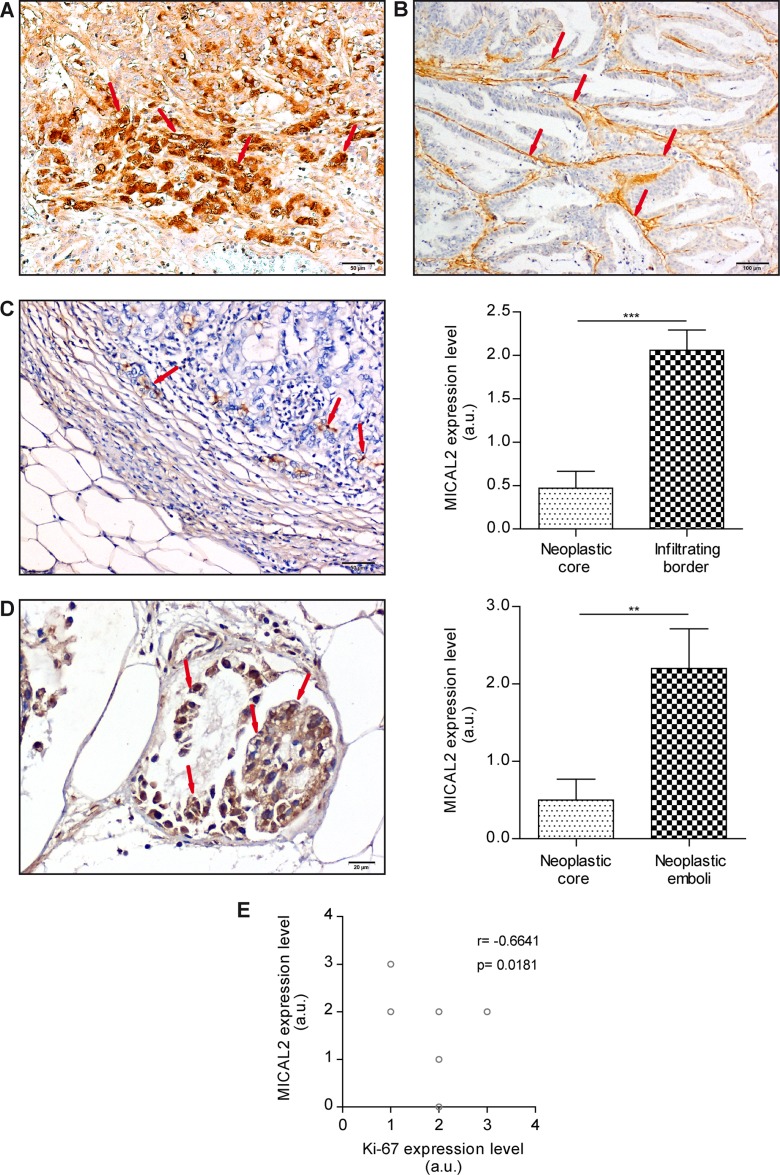
MICAL2 protein is abnormally expressed in primary human GC (IHC analysis) (**A**) Strong MICAL2-positive immunodecoration of the tumor, mostly as scattered neoplastic cells infiltrating the gastric wall (DAB stain, arrows). Scale bar: 50 μm. (**B**) Intestinal-type tumors showed very low to undetectable MICAL2 expression in cancer cells, but strong immunodecoration of neo-angiogenic ECs (DAB staining, arrows). Scale bar: 100 μm. (**C**) MICAL2-positive cells (arrows) were found at the tumor infiltrating border rather than in the neoplastic core. Scale bar: 50 μm. MICAL2 mean expression level at the tumor infiltrating edge is higher than in the neoplastic core, *N* = 17, ***p ≤ 0.001 (Student's *T* test for paired samples). (**D**) MICAL2-positive cells in neoplastic emboli (8/10, 80%). Scale bar: 20 μm. MICAL2 expression in emboli was higher than in the neoplastic core, *N* = 10, **p ≤ 0.01 (Student's *T* test for paired samples). (**E**) Qualitative IHC analysis (images not shown) of single immunolabelling indicated Ki-67 expression level inversely correlated with MICAL2 expression. *N* = 10, *r* = −0.6641; *p* = 0.0181 (Pearson correlation test).

In GC, IHC showed a patchy distribution of MICAL2 over-expression in comparison with normal surrounding mucosa in 31/40 samples (77.5%). In diffuse, signet ring adenocarcinomas, MICAL2 expression was high within the tumor mass and in scattered neoplastic cells infiltrating the gastric wall (Figure 2A). Conversely, MICAL2 expression was undetectable in cancer cells of well differentiated, intestinal-type tumors (Figure 2B). MICAL2-positive cells were localized at the tumor infiltrating front (17/17 samples, 100%, Figure 2C left) rather than within the neoplastic core (*p* = 0.0001, Figure 2C right). Moreover, MICAL2-positive cells were found in neoplastic emboli (8/10 samples, 80%, Figure 2D left), where MICAL2 expression was higher than in the neoplastic core (*p* = 0.0038, Figure 2D right), and where sometimes it showed both nuclear and cytoplasmic localization. When variable differentiation grades were present within the same tumor, MICAL2 immunolabelling was higher in the less differentiated areas. Endothelial cells (ECs) of neo-angiogenic capillaries that extensively branch into the cancer mass showed a very intense MICAL2 immunostaining (Figure 2B), but no expression of MICAL1 and MICAL3 (Supplementary Figure 2), suggesting a different role of MICAL proteins within neo-angiogenic ECs.

Given the peculiar localization of MICAL2-positive cells on the cancer invasive front, we wondered how this would correlate with the tumor cells proliferative state. Therefore, we analyzed the expression of Ki-67, a ubiquitous cell proliferation marker [18]. Consecutive sections from the same paraffin blocks were used for either immunostaining of MICAL2 or Ki-67. We found Ki-67 expression level inversely correlated with MICAL2 expression (*p* < 0.05, Figure 2E).

**Figure 3:**
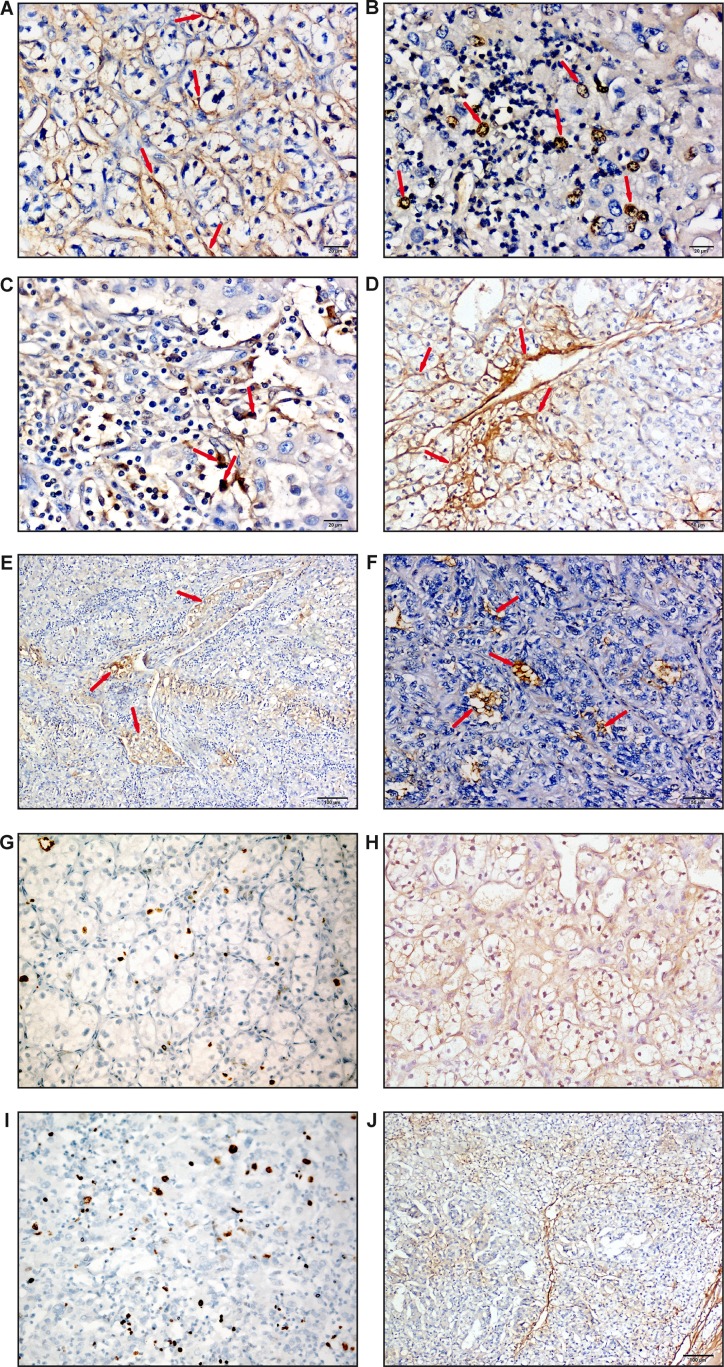
MICAL2 is abnormally expressed in human kidney cancer IHC analyses (DAB stain, arrows) showed MICAL2 subcellular localization was (**A**) either cytoplasmic, (**B**) nuclear, (**C**) or both, scale bar: 20 μm. (**D**) Also in kidney cancer, MICAL2 was highly expressed in neo-angiogenic ECs. Scale bar: 50 μm. (**E**), Neoplastic emboli of metastases to the lung showed cells with intense MICAL2-positive immunodecoration (Scale bar: 100 μm), however (**F**) in the metastatic mass MICAL2 immunolabeling was very low and limited to rare, scattered peripheral cells (Scale bar: 50 μm). Representative images of cancer areas in which: (**G**) low expression of Ki-67 was associated with (**H**) relatively high expression of MICAL2 (sample ID: 10190), and (**I**) relatively high expression of Ki-67 with (**J**) low/undetectable expression of MICAL2 in cancer cells, with expression localized to interstitium as evident from low magnification image (sample ID: 14587).

In ccRCC, the subcellular localization of MICAL2 was either cytoplasmic (Figure 3A), nuclear (Figure 3B), or both (Figure 3C) with cellular pattern variability within the same tumor. In addition, MICAL2 was highly expressed in ECs of the numerous cancer-associated capillaries (Figure 3D). Noticeably, MICAL2-positive neoplastic cells were detected within tumor emboli of lung metastases (Figure 3E) whereas, in the remaining cells of metastatic lesions to the brain (not shown) and lung (Figure 3F), MICAL2 protein expression was very low, localized to rare and scattered peripheral cells within the metastatic mass. MICAL2 protein was low in normal tubules and undetectable in normal glomeruli (Supplementary Figure 3).

Although it was not feasible to evaluate systematically and quantitatively the cancer invasive edge in the ccRCC samples available for this study, qualitative IHC analysis of single-antigen immunolabelling showed Ki-67 (Figure 3G) to be mainly expressed in the tumor core where MICAL2 (Figure 3H) was not or scarcely expressed, and vice versa (Figure 3I, 3J). So, MICAL2 expression inversely correlated with proliferation also in kidney cancer.

Altogether, data showed MICAL2 expression was high in aggressive primary cancer and in metastatic emboli, but barely or not detectable in cancer cells at metastatic sites.

### Depletion of MICAL2 in cancer cells induces MET *in vitro*

With knock-down experiments, we investigated *MICAL2* loss-of-function in human cancer cell lines with mesenchymal phenotype derived from kidney (786-O), breast (MDA-MB-231) and pleural cavity (MERO-14) respectively (generated as in Supplementary Figure 4). We performed RNA interference with commercial siRNA-expressing plasmids, using three specific siRNAs for the target of interest, plus one non-specific siRNA and the corresponding empty vector as references. All through the text, cell lines are designated as follows: parental, untreated cancer cell lines as ‘WT’; cells transfected with control plasmid (either TRCN0000192634 or empty pLKO.1-Neo-CMV-tGFP) as ‘CTRL’ cells; 786-O MIC2-KD clones as KD2 (obtained from tranfection with a single plasmid, TRCN0000046581) and KD14 (obtained from simultaneus transfection with three plasmids plasmids TRCN0000046579, TRCN0000046580 e TRCN0000046581); MERO-14 MIC2-KD cells as KDM1; MDA-MB-231 MIC2-KD cells as KDM4. (KDM1 and KDM4 were obtained from transfection with all three plasmids). CTRL and parental cells are collectivey referred to as ‘reference’ cells. For details, see Materials and Methods and Supplementary Materials.

**Figure 4:**
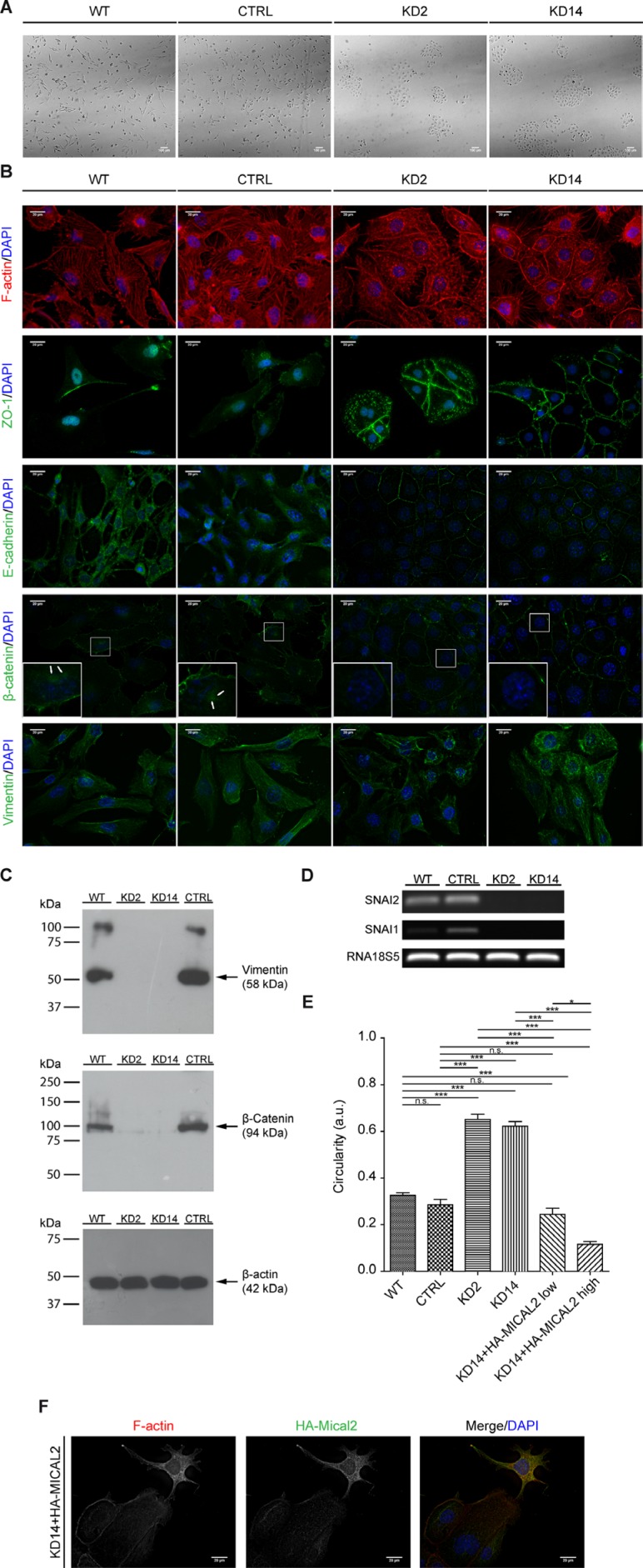
Abating *MICAL2* in 786-O kidney cancer cells *in vitro* induces MET (**A**), Morphological analysis in light-transmitted microscopy. Scale bar: 100 μm. (**B**), F-actin staining. IF of: E-cadherin, ZO-1, catenin-beta. In KD2 and KD14 cells, these three markers relocated at cell perimeters (as in epithelial cells), rather than being diffusedly cytoplasmic. IF of vimentin with Vim13.2 antibody. In MIC2-KD cells it decorated short and disorganized filaments instead of long meshes observed in control cells. Scale bar of all IF images: 20 μm. Inset magnification: 3x. (**C**), Vimentin epitope recognized by V9 antibody was undetectable in KD2 and KD14 cells. (**D**), Agarose gel run of SNAI1, SNAI2, 18s5 RT-PCR products. (**E**), KD14 cells transfected with HA-MICAL2 cDNA. The elongated phenotype showed dose-dependency on HA-MICAL2 expression. One-way Anova test and Tukey's Multiple Comparison post-hoc test. In all graph bars, horizontal lines denote mean and SEM. N.s: non significant. *p ≤ 0.05, **p ≤ 0.01, ***p ≤ 0.001). (**F**), MIC2-KD14 cells expressing HA-MICAL2 (shown by anti-HA immunostaining) recovered a mesenchymal-like phenotype with concomitant F-actin redistribution.

786-O MIC2-KD cells immediately appeared morphologically different (Figure 4), regardless of cell density in the culture plate (not shown). Instead of the elongated, mesenchymal-like shape and scattered distribution on the plate, with minimal if any intercellular contacts, MIC2-KD cells appeared larger, organized in tightly packed clusters, with apico-basal polarity of epithelial cells (Figure 4A).

Through its redox activity, D-MICAL is a direct regulator of F-actin dynamics in neural cells [4]. In non-neural cells, human MICALs are also regulators of actin stress fibers [13]. We stained F-actin to further characterize MIC2-KD phenotype, and observed a clearly different F-actin distribution (Figure 4B). Circumferential actin belts of individual cells, with abundant intracellular puncta, loss of prominent stress fibers, and presence of well-defined rims, indicative of cell-cell junctions in polarized cells of epithelial sheets [19], were visible in MIC2-KD cells, whereas reference cells (CTRL and parental) presented stress fibers and scarse pericellular F-actin, in agreement with [13].

To test the hypothesis that *MICAL2* down-regulation leads to MET, we performed immunocytochemistry (ICC) analysis of typical epithelial markers of cell-cell contact, such as Zonula Occludens 1 (ZO-1) at tight junctions, E-cadherin and catenin-beta (CTNNB1) at adherens junctions. These markers were found sharply localized at cell-cell junctions in MIC2-KD cells, designing finely honed cell perimeters, typical of epithelial cells, rather than diffusely distributed in the cytoplasm as in mesenchymal-like reference cells (Figure 4B). Also, MIC2-KD cells showed short and disorganized vimentin filaments, compared with the longer meshes observed in control cells (Figure 4B). Vimentin and CTNNB1 expression was analyzed also by Western blot (WB). In MIC2-KD cells, CTNNB1 level was found strongly decreased (Figure 4C); WB anti-vimentin performed with V9 antibody (an anti-vimentin antibody different from that used for IF) did not yield any signal, as if the corresponding epitope was undetectable.

Signaling pathways activated by intrinsic or extrinsic stimuli responsible for eliciting EMT/MET converge on transcription factors (TFs) ultimately regulating the phenotypic changes, like Snail family zinc finger (SNAI)1 and SNAI2 that impart mesenchymal traits to tumor cells including increased motility and invasiveness, and are responsible for transcriptional repression of E-cadherin [20, 21]. With QRT-PCR the expression of both SNAI1 and SNAI2 resulted undetectable in KD2 and KD14 cells (Figure 4D).

Further, over-expression of *MICAL2*-tagged cDNA (HA-MICAL2) in flat, cobblestone-like, MICAL2-depleted, KD14 cells produced motile, fibroblast-like cells (Figure 4E, 4F), resulting in the rescue of the mesenchymal phenotype of parental 786-O cells. To describe the cell shape change, we performed a quantitative morphometric analysis of cell perimeter, and characterized cell shape with circularity analysis. 786-O reference cells showed a more elongated phenotype (mean circularity values of 0.326 and 0.285 respectively) compared with the two MIC2-KD cell lines (mean circularity values of 0.651 for KD2 and 0.621 for KD14, Figure 4E). Dividing the population of transfected MIC2-KD14 cells into two groups of low-fluorescence and high-fluorescence cells (HA-MICAL2^low^ and HA-MICAL2^high^) as monitored by IF with anti HA-tag antibody, we also noticed a more elongated phenotype with dose-dependency on HA-MICAL2 expression (mean circularity values of 0.244 for HA-MICAL2^low^ and 0.116 for HA-MICAL2^high^, Figure 4E).

### Knocking-down *MICAL2* reduces cell viability, adhesion and motility on 2D surfaces, and invasion properties in 3D matrices of cancer cells *in vitro*

**Figure 5:**
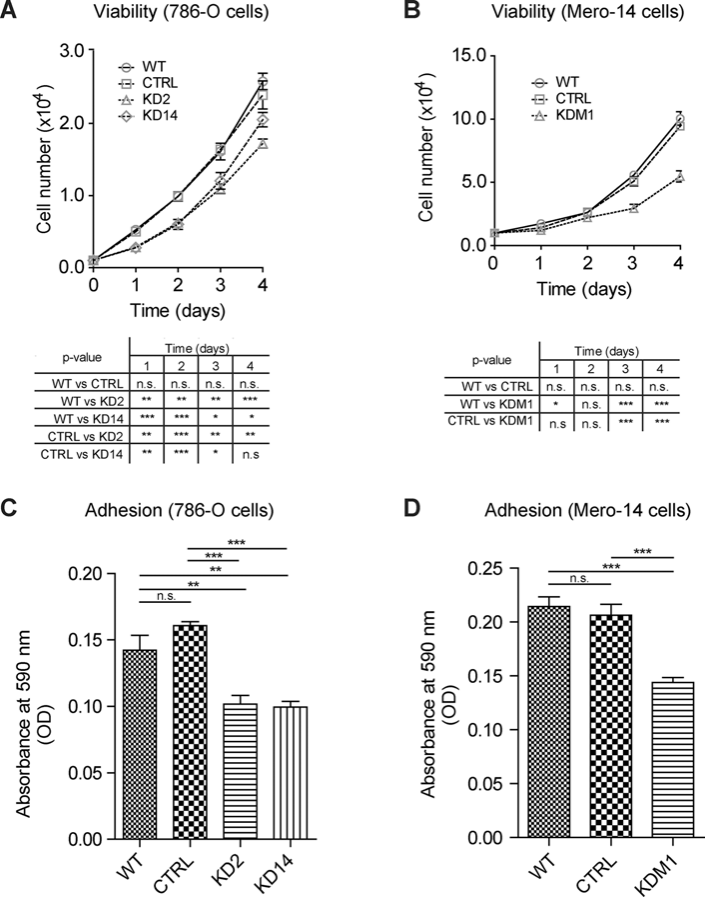
Depleting *MICAL2* reduces cancer cells viability and adhesion *in vitro* MIC2-KD cells showed significantly decreased viability compared with respective (**A**) 786-O and (**B**) MERO-14 WT and CTRL cells. MIC2-KD reduced adhesion of KD2 and KD14 (33%, (**C**) and KDM1 (30%, (**D**) cells on type-I collagen. One-way Anova test and Tukey's Multiple Comparison post-hoc test. In all graph bars, horizontal lines denote mean and SEM. N.s.: non significant. *p ≤ 0.05, **p ≤ 0.01, ***p ≤ 0.001.

To investigate how MIC2-KD affected basic cellular mechanisms like proliferation, cell adhesion, migration and invasion, we performed viability and functional assays in 786-O, MDA-MB-231 and MERO-14 cells. MTT-based cell-growth assays and manual counting showed that reducing *MICAL2* mRNA level significantly decreased 786-O and MERO-14 cell viability (Figure 5A, 5B). Since cell adhesion and motility play a key role during EMT, and MICAL2 has been implicated in growth cone dynamics [3, 9], we asked whether MICAL2 expression might affect cancer cell adhesion *in vitro*. On type-I collagen-coated plates, 786-O KD2 and KD14 cells showed 33% reduction in adhesion capacity (Figure 5C), and similar results were obtained with MERO-14 KDM1 cells (reduction of 30%, Figure 5D).

**Figure 6:**
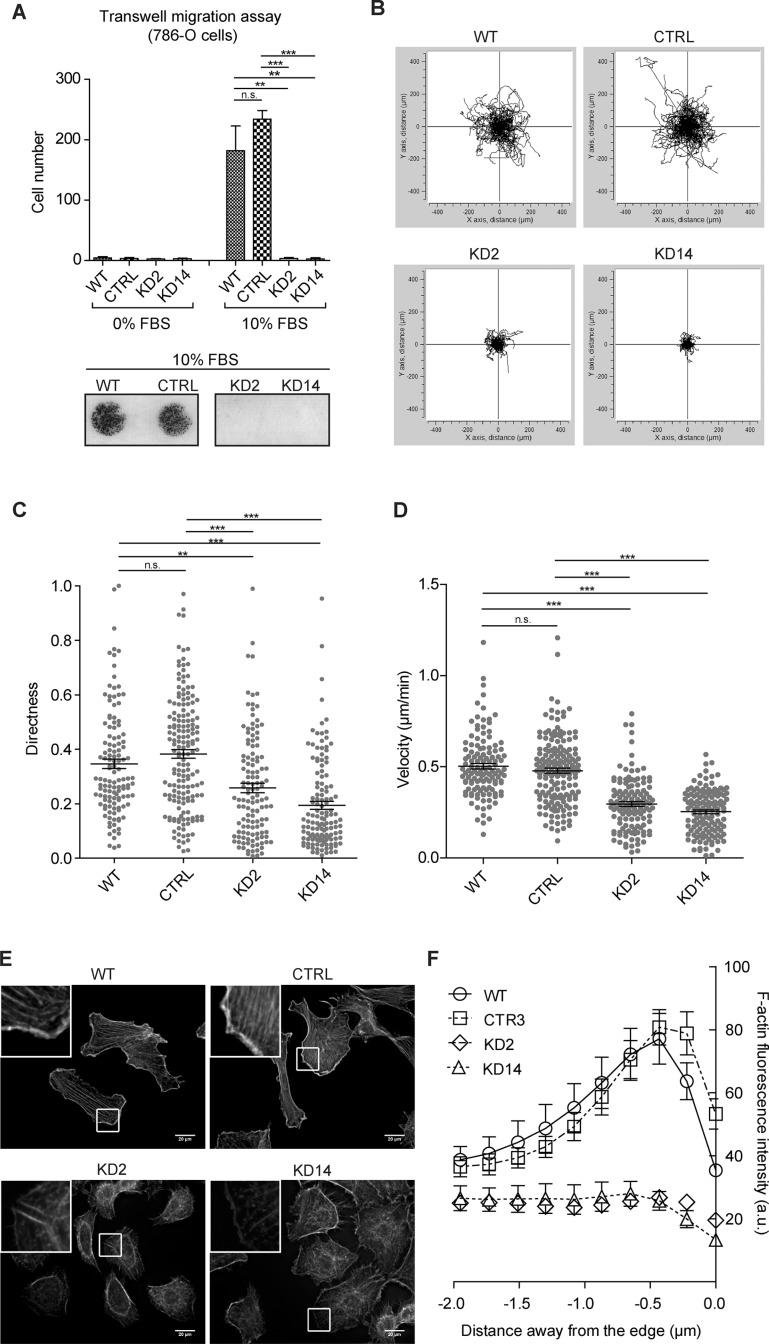
*MICAL2* abatement *in vitro* reduces motility of 786-O ccRCC cells (**A**) Transwell migration assay: MIC2-KD cells were not able to cross the 10 μm-pore membranes of blind-well chambers, even in the presence of 10% FBS. Lower panel: result of a representative experiment. (**B**) Single-cell traces of 15-hr chemokinesis assay: KD2 and KD14 cells run an extremely short distance. Directness (**C**) and Velocity (**D**), indicators of oriented movement, were reduced. (**E**) (**F**)-actin staining with Phalloidin. Scale bar: 20 μm. Inset magnification: 3.7X. F, Fluorescence intensity derived from Phalloidin staining is measured for 2 μm across the cell border; negative distances are meant from the outer side of the contour toward the inner cell side. The peculiar peak of fluorescence intensity, occurring within the first μm away from the leading edge of lamellipodia in reference cells, was lost in KD2 and KD14 cells. A, C and D: One-way Anova test and Tukey's Multiple Comparison post-hoc test. In all graphs, horizontal lines denote mean and SEM. N.s.: non significant. *p ≤ 0.05, **p ≤ 0.01, ***p ≤ 0.001.

Further, a chemotaxis assay showed KD2 and KD14 cells were not able to cross the blind-well chamber membrane, even in the presence of 10% FBS (Figure 6A). We also performed a random 2D migration (chemokinesis) assay by plating cells in the presence of a diffuse chemokinetic stimulating agent (10% FBS). Time-lapse microscopy showed persistent migration by 786-O reference cells when observing single-cell traces obtained during the 15 hr experiments, while KD2 and KD14 cells almost did not leave the origin (Figure 6B and Supplementary Movies). MIC2-KD cells moved randomly (Figure 6C) and more slowly (Figure 6D). We also observed a different behavior of the lamellipodia (Figure 6E, 6F). Reference cells migrated by extending dynamic protrusions with polarized and persistent lamellipodia, well-defined trailing and leading edges. In contrast, most MIC2-KD cells showed dynamic but irregularly shaped lamellipodia, often broader, almost circular, from which multiple, small, narrow and highly dynamic protrusions sticked out (Supplementary Movies and Figure 6E). This quasi radial ‘symmetrization’ of the cell motility apparatus resembled closely the ‘stop signal’ caused by prolonged contact [22], although in this case at the presence of very few surrounding cells. These results suggested *MICAL2* abatement changed cell membrane protrusion morphology and behavior, and consequently net cell movement in 2D migration.

Since several primary carcinoma specimens showed MICAL2-positive cells on the cancer invasive edge and in intravasal tumor emboli (Figure 2C, 2D), we tested the contribution of MICAL2 to invasive properties of cancer cells *in vitro*. We performed invasion assays with 786-O and MDA-MB-231 cells in 3D collagen matrix to characterize the migratory properties of cancer cells challenged to overcome the stiffness inherent to the surrounding matrix, a condition absent in 2D migration.

**Figure 7:**
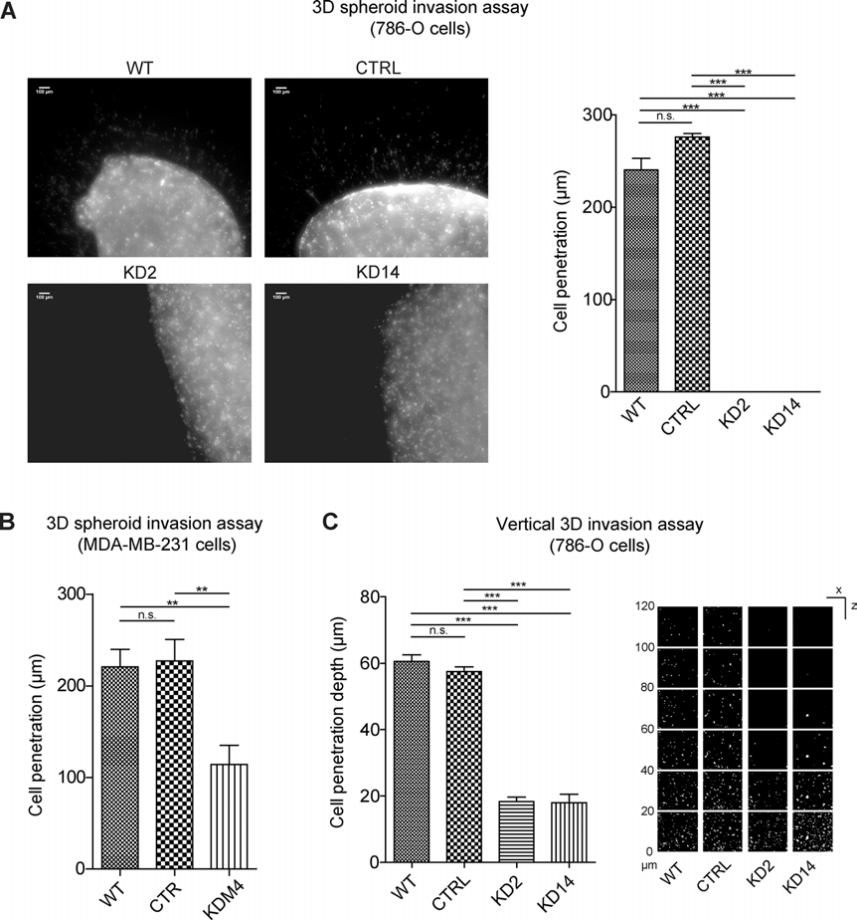
MICAL2 abatement strongly reduces cancer cell 3D invasion properties (**A**) Representative result of 3D spheroid invasion assay. (**B**) KDM4 showed reduced invasion activity. (**C**) Right: representative result of vertical 3D assays. One-way Anova test and Tukey's Multiple Comparison post-hoc test. In all graphs, horizontal lines denote mean and SEM. N.s.: non significant. *p ≤ 0, 05, **p ≤ 0, 01, ***p ≤ 0, 001.

In 3D spheroid invasion assay, kidney cancer KD2 and KD14 cells were totally unable to invade the surrounding matrix during the 72 hr-experiment. Breast cancer cells KDM4 also showed reduction of invasion capacity (Figure 7B).

In vertical 3D invasion assay, 786-O MIC2-KD cells run only 33% of the average distance of cell migration above 20 μm performed by reference cells (Figure 7C). Altogether, these results suggested MICAL2 activity is necessary to confer cells the ability to detach from an origin and move within a complex 3D matrix.

### MICAL2 is implicated in radical oxygen species (ROS) production in cancer cells

**Figure 8:**
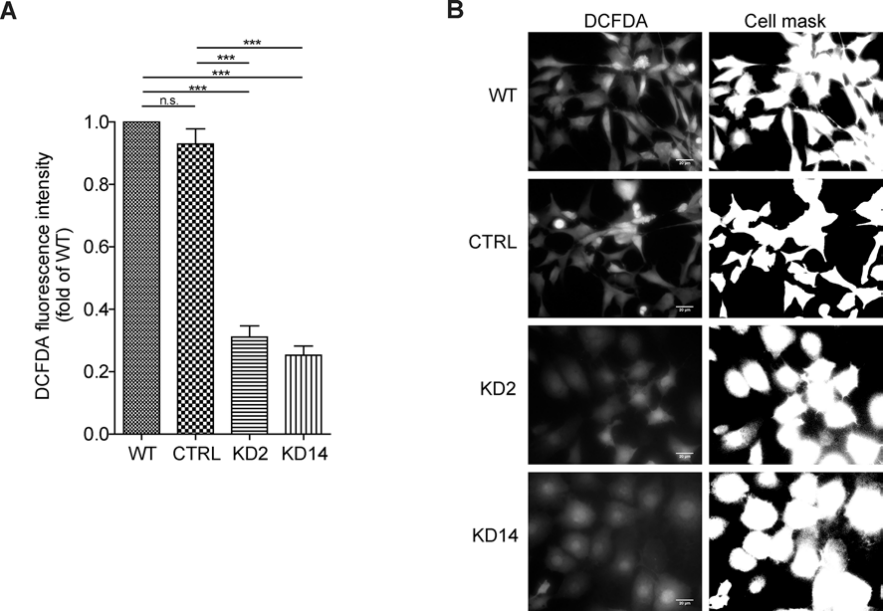
MICAL2 is implicated in radical oxygen species (ROS) production in cancer cells (**A**) Detectable ROS levels were present in WT and CTRL cells, by contrast a three to four folds reduction in H_2_DCF fluorescence in the MIC2-KD clonal lines was found. (One-way Anova test and Tukey's Multiple Comparison post-hoc test. Horizontal lines denote mean and SEM. Ns: non significant. *p ≤ 0, 05, **p ≤ 0, 01, ***p ≤ 0, 001). (**B**) representative micrographs showing H_2_DCF fluorescence in 786-O WT, CTRL, KD2 and KD14 cells (left) and binary mask created in ImageJ by applying a threshold, to visualize cell boundaries (right).

MICAL proteins display an N-terminal MO domain which binds flavin-adenine-dinucleotide (FAD) and uses the coenzyme nicotinamide-adenine-dinucleotide-phosphate (NADPH) in redox reactions to either directly oxidize substrate proteins and signaling molecules and/or produce ROS [5, 7, 8]. ROS in general and H_2_O_2_ in particular were associated with EMT induction and sustenance [23]. Therefore, we tested the hypothesis that reducing MICAL2 might affect ROS production. 786-O WT, CTRL, KD2 and KD14 cells were treated for 30 minutes with 25 uM 2′, 7′-dichlorodihydrofluorescein diacetate (H_2_DCFDA), a cell-permeant ROS probe which is converted in the fluorescent 2′, 7′-dichlorodihydrofluorescein (H_2_DCF) upon oxidation within the cell. Cells were then live imaged with a fluorescent microscope to detect H_2_DCF fluorescence (Figure 8). A three to four folds H_2_DCF fluorescence reduction was found in MIC2-KD clonal lines.

We concluded that MICAL2 activity participates to ROS production, which is in agreement with current literature showing MICAL2-dependent ROS level increase in HeLa cells over-expressing MICAL2 [13].

## DISCUSSION

To search for novel metastatic factors, we investigated a possible role in cancer for MICAL2, a protein involved in controlling cytoskeleton plasticity, a trait extremely relevant to cell rearrangements and motility in metastatic dissemination.

We found MICAL2 significantly over-expressed in poorly differentiated and undifferentiated gastric and kidney cancer, compared with more differentiated carcinomas. In ccRCC patients, MICAL2 over-expression was significantly associated with metastasis (Figure 1).

All observations in primary and metastatic human tumors suggested that *MICAL2* expression is ‘on’ in a subpopulation of primary cancer cells seemingly detaching from the origin and prone to actively migrate, in individual mode, resist anoikis and travel to distant sites. In brief, are in a state of EMT. In fact, recent data show that MICAL2 is part of a gene signature that specifically characterize high-grade tumors bearing mesenchymal markers [24]. MICAL2 is then turned ‘off’ following homing at the metastatic site, where MET occurs. The hypothesis of an ‘on-off’ expression mode during cancer progression, sustained by our *in vitro* functional analysis, is in line with current literature supporting that cancer spreading depends on mesenchymal-like cells located on the cancer invasive front [10, 25].

This matches well with previous studies showing that loss of epithelial markers, like E-cadherin, and gain of mesenchymal markers, like vimentin (both events observed in our *in vitro* studies) can promote intravasation and early steps of metastasis, but then re-epithelialization is necessary for proliferation of disseminated cells at metastatic sites [26, 27].

Also the inverse correlation with Ki-67 expression level suggested that MICAL2 expression might occur in relatively few (compared with the tumor mass) but critical cells endowed with a motile, invasive phenotype at the tumor edge, rather than proliferating *in situ*.

So MICAL2 is probably turned on by specific stimuli in the cancer microenvironment and its expression, after having accompanied/sustained cancer cell migration, is turned off at the metastatic site where the intensity of those signals has faded. This allows MET and reprise of proliferation. Consistently, MIC2-KD cells showed *in vitro* a coherent image of MET revealed by the dramatic reorganization of F-actin and by molecular analysis of indicative markers [28, 29] such as E-cadherin, ZO-1, CTNNB1 and vimentin (Figure 4B), suggesting indirectly that MICAL2 regulated function is necessary for formation of proper cell-cell junctions. Interestingly, vimentin and CTNNB1 over-expression and/or cytoplasmic accumulation (as we found in cancer cells expressing high level of MICAL2 *in vitro*) are both known predictors of hematogenous metastasis in human cancer [30, 31].

MET in MIC2-KD cells occurred through transcriptional reprogramming, indicated by the decrease/loss of expression of *SNAI1* and *SNAI2* (Figure 4D), two TFs crucial in initiating/maintaining EMT [19–21]. How this happens was not addressed in this work but, based on information about other MICAL family members [14, 32–36], MICAL2 might take part in vesicular trafficking required for transfer of cell surface receptors or other cell signaling event.

Furthermore, when we reconstituted *MICAL2* expression by cDNA transfection in MIC2-KD cells, that have epithelioid phenotype, we obtained a mesenchymal phenotype (Figure 4E, 4F). On one hand this supported the specificity of the results of our knock-down approach and, on the other, more importantly, suggested that the expression/re-expression of MICAL2 is sufficient to induce EMT in epithelial cells.

Overall, all MIC2-KD cell populations generated from cancer cells of different histotypes were found less adherent (Figure 5) and noticeably less capable of migrating (Figure 6) and invading (Figure 7) in 2D and 3D assays *in vitro*, consistently with a MET phenotype. Directional migration requires cell polarity, but MIC2-KD cells lacked a clearly polarized arrangement in 2D and 3D assays, suggesting that in cancer MICAL2 endogenous over-expression might promote invasion. Often, in metastatic cancers other actin-modifying enzymes were found expressed in deregulated ways. Although that is not a cancer-initiating event it can certainly be a cancer-promoting factor, because expression normalization can revert the metastatic phenotype [38].

Finally, it was proposed that in addition to directly modifying F-actin through redox reactions, MICALs may influence intracellular signaling through generation of ROS as second messengers, in a physiologically restricted manner [9]. ROS have *per se* notorious pro-metastatic, pro-neoangiogenic effects. Our data showing sharp decrease of ROS production in MIC2-KD cells suggest that endogenous over-expression of MICAL2 in human cancer might generate high ROS level with a consequent cell-autonomous, pro-metastatic, pro-angiogenic effect during EMT. MIC2-KD might contribute to ROS decrease and MET also through inhibition of SNAI1, whose expression increases ROS level in cancer cells leading to EMT [39].

In conclusion, this is the first work to show data from human cancer and *in vitro* analyses suggesting that MICAL2 represents a marker of metastatic disease that promotes migration and invasion of epithelial cancers. MICAL2 might represent a new therapeutic target for thwarting epithelial cancer cell pro-invasive and pro-metastatic potential.

## MATERIALS AND METHODS

### Tissue specimens

Human paired tumor/normal tissue samples were retrospectively obtained from patients who underwent surgery with curative or palliative intent for primary gastric cancer (*N* = 64) at the Department of Pathological Anatomy of Azienda Ospedaliera Universitaria Senese (Siena, Italy) and the Department of General Surgery and Oncology (University of Siena, Italy), and for lung (*N* = 27 NSCLC) and kidney cancer (*N* = 36 ccRCC) at the Blokhin Cancer Research Center (Russian Academy of Medical Sciences, Moscow, Russia). The study was done in accordance with the Declaration of Helsinki. Approval was obtained from pertinent ethics committee. All patients gave informed written consent. Patients who had undergone pre-operative radiotherapy or chemotherapy were excluded. Samples were taken in the operating room and stored in liquid nitrogen. In addition to the tumor, a sample of paired normal tissue was collected from the operative specimens at least 10 cm from the tumor, when applicable. Only samples containing 70–80% or more tumor cells, and adjacent specimens, fixed in formalin and embedded in paraffin, were used.

Diagnosis and histological grading were assessed using standard criteria by experienced pathologists. Renal cell carcinomas were classified and graded according to [17] and staged according to [40]. Gastric cancers were classified in intestinal, diffuse and mixed histotype according to [16] and staged according to [40].

### Cell lines and culturing

786-O (ATCC CRL-1932), and MDA-MB-231 (ATCC HTB-26) were purchased from ATCC-LGC Standards (Eu), MERO-14 from Sigma.

Cell lines were purchased *ad hoc* for the study and kept in appropriate growth medium additioned with 100 IU/ml penicillin, 100 μg/ml streptomycine: 786-O in RPMI-1640 (Sigma-Aldrich) with 10% heat-inactivated fetal bovine serum (FBS, Gibco), 10 mM HEPES, 1 mM Na pyruvate, 100 mM glutamine; MDA-MB-231 in RPMI-1640 (Sigma-Aldrich) with 10% FBS (Gibco), 100 mM glutamine; MERO-14 in DMEM (Sigma-Aldrich) with 15% FBS (Gibco), 200 mM glutamine. All cell cultures were kept in 5% CO_2_ incubator at 37°C, and routinely tested for mycoplasma contamination (MycoAlert Kit, Lonza).

### *In silico* analysis

Public bioinformatic resources used for this study: http://www.ncbi.nlm.nih.gov/unigene/, http://www.cbs.dtu.dk/services/NetPhos/, http://web.expasy.org/findmod/, http://rsbweb.nih.gov/ij/index.html, http://www.genesapiens.org/, http://discover.nci.nih.gov/cellminer/analysis.do.

### RNA and cDNA preparation for retrotranscription-PCR (RT-PCR) and semi-quantitative real time-PCR (QRT-PCR)

Total RNA was isolated with RNeasy Mini Kit (Qiagen). Purified total RNA samples were quantified spectrophotometrically (NanoDrop ND-1000, NanoDrop Technologies, USA), and quality-checked (Bioanalyzer 2100, Agilent Technologies, USA). cDNA was synthesized using random hexamers and oligo-dT primers (QuantiTect Reverse Transcription Kit, Qiagen).

RT-PCR was performed in 2720 Thermal Cycler (Applied Biosystems, USA), with GoTaq Flexi DNA Polymerase (Promega). QRT-PCR was performed in technical and when applicable biological triplicates, in ABI 7000 PRISM SDS thermal cycler (Applied Biosystems). Data were analyzed through relative quantification with the −ΔΔCt method.

Sequence of primers: EEF1A1 (NM_001402.5): 5′-CTTTGGGTCGCTTTGCTGTT-3′, 5′-CCGTTCTTC CACCACTGATT-3′; GAPDH (NM_001289746.1); 5′-GC TCATTTCCTGGTATGACAACG-3′, 5′-AGGGGTCTAC ATGGCAACTG-3′; beta-actin (NM_001101.3): 5′-AACT GGAACGGTGAAGGTGACAGC-3′, 5′-AGAAGTGG GGTGGCTTTTAGG-3′; PPIA (NM_021130.3): 5′-TTC ATCTGCACTGCCAAGAC-3′, 5′-TCGAGTTGTCCACA GTCAGC-3′; 18S5 (NR_003286.2): 5′-GTAACCCGT TGAACCCCATT-3′, 5′-CCATCCAATCGGTAGTAGC G-3′; MICAL2 (NM_014632.2) : 5′-CAACCCGTGT GTGTCTCATC-3′, 5′-GTGGATGCCTGGACAAAGT T-3′; SNAI1 (NM_005985.3): 5′-AATCGGAAGCCTA ACTACAGCG-3′, 5′-GTCCCAGATGAGCATTGGCA-3′; SNAI2 (NM_003068.4): 5′-GACCCTGGTTGCTTCA AGGA-3′, 5′-TGTTGCAGTGAGGGCAAGAA-3′.

Primers were used at the following conditions: 2 min at 95°C; 33 cycles of 30 sec at 95°C, 30 sec at 58°C, 15 sec at 72°C; 5 min at 72°C. Amplicons were sequence-verified (3730 DNA Analyzer automated sequencer, Applied Biosystems).

For QRT-PCR on human cancer specimens, Hs01121791_m1 (human MICAL2) and Hs01111406_g1 (human RPN1) TaqMan GeneSpecific Assays FAM dye-labeled (Applied Biosystems) and TaqMan Universal PCR Master Mix (Applied Biosystems) were used. Human RPN1 was used as reference gene. RPN1 mRNA level variability was not higher than 2-fold in tumor (T) and normal (N) tissues. The reference gene for QRT-PCR on cancer cell lines was 18S5.

### Western blotting, immunofluorescence, immunohistochemistry

Western blotting (WB) was performed according to standard protocols. Immunofluorescence (IF) analysis was performed as in [41]. Immunohistochemistry (IHC) was performed as in [42]. ICC with DAB staining was performed as in [43].

Cells fixed for IF analysis were incubated with primary and secondary antibody solutions, DAPI (Sigma-Aldrich), and mounted in Aqua PolyMount (Polysciences, USA).

IHC was performed as in [42] with avidin-biotin complex technique on paraffin embedded sections rehydrated and treated with H_2_O_2_ before incubation with primary and secondary antibody solutions, and pertinent substrate (CN/DAB Substrate Kit, Thermo Scientific), and finally mounted in Canada Balm (Bioptika).

Primary antibodies for WB: rabbit polyclonal anti-MICAL2 (1:1000, generated and validated in our laboratory as in [44], see Supplementary Figure 1), anti-CTNNB1 (1:1000, Thermo Scientific, USA), anti-vimentin V9 (1:5000, Sigma), anti-beta-actin (1:7000, Sigma-Aldrich). Secondary antibodies: HRP-conjugated goat anti-rabbit (Pierce); HRP-conjugated goat anti-mouse (Pierce).

Primary antibodies for IF: rabbit polyclonal anti-MICAL2 (1:1000, generated and validated in our laboratory as in [44], see Supplementary Figure 1), anti-E-caderin PA5–27187 (1:100 Thermo Scientific, USA), anti-ZO-1 40–2300 N-term (1:500, Invitrogen, USA), anti-HA (1:200, Sigma), anti-CTNNB1 (1:100, Thermo Scientific, USA), anti-vimentin VIM 13.2 (1:500, Sigma). Secondary antibodies: Goat anti-rabbit Alexa Fluor 488 (and 568, not shown): 1:1000 (Molecular Probes, USA). Rabbit anti-mouse Alexa Fluor 568: 1:1000 (Molecular Probes, USA).

Primary antibodies for IHC: rabbit polyclonal anti-MICAL2 (1:2500 or 1:1500, generated in our laboratory as in [44], see Supplementary Figure 1), anti Ki-67 (Novocastra Reagents, Leica Biosystems, Australia), anti-MICAL1 (1:100, Santa Cruz, USA), anti-MICAL3 (1:100, Santa Cruz, USA). Secondary antibodies provided by VECTASTAIN Elite ABC Kit (Vector Laboratories, USA).

C.V. and A.P. evaluated the results. The immunoreaction was evaluated in arbitrary units (a.u.) in a range from 0 a.u. to 4 a.u. on the bases of antigen-positive cell percentage in the region of interest (0% = 0 a.u.; 1%–25% = 1 a.u.; 26%–50% = 2 a.u.; 51%–75% = 3 a.u.; 76%–100% = 4 a.u.).

### Staining of F-actin

F-actin was stained with 488 Acti-stain and 568 Phalloidin (Cytoskeleton).

### Cell circularity

After tracing manually the cell perimeters, cell circularity was measured using the circularity plugin built in ImageJ, according to the equation: Circularity *C* = 4π*A*/*P*^2^ (*P* = cell perimeter, *A* = cell area).

### Cell viability assay

Cell proliferation and viability were tested with manual counting and/or with colorimetric assay based on 3-(4, 5-Dimethylthiazol-2-yl)-2, 5-diphenyltetrazolium bromide (MTT, Sigma-Aldrich), starting from 10^3^ cells, for 4 days. OD values at 590 nm (ETI-SYSTEM Fast Reader, Sorin Biomedica, Italy) were plotted against a cell line-specific reference curve to infer the corresponding cell number.

### 2D cell adhesion and motility assays

Adhesion was evaluated according to standard protocols by plating 10^4^ cells on vessels coated with 0.1 μg/μl type-I collagen (Sigma-Aldrich). Cells were challenged after 1 hr (786-O) or 15 minutes (MERO-14) from plating, then colored with crystal-violet solution and read at 590 nm (ETI System Fast Reader, Sorin Biomedica, Italy).

Chemotaxis assay was performed with a modified Boyden Chamber mounting 10 μm polycarbonate membrane (NeuroProbe, USA). 8 × 10^3^ cells were seeded in the upper chamber with serum-free growth medium and incubated for 16 hrs. The lower chamber contained growth medium plus 10% FBS (or no FBS for negative control). Staining was performed with Diff-Quick (Medion Diagnostics, Switzerland).

For chemokinesis assay, 2 × 10^3^ cells were seeded in cell growth medium with 10% FBS. Live imaging was performed (15 hrs) with a microscope-fitting a custom-made 5% CO_2_ incubation chamber. Directness represents a measure of the cell's tendency to travel in a straight line. It is calculated as the ratio of the Euclidian distance and the accumulated distance in time: *D* = *d*_E_/*d*_A_. *D* = 1 indicates a straight-line migration between start and end points [45].

Velocity is the ratio between the accumulated distance and time (experiment duration): *V* = *d*_A_/*T*, where *T* = total experiment time.

### 3D invasion assays

3D spheroid invasion assay and 3D vertical assay were performed as in [46] starting with 5 × 10^4^ cells embedded in droplets of RPMI-1640 (Sigma-Aldrich) and 2.2 mg/ml type-I collagen (BD Biosciences), at pH 7.4. Z-stack images were captured every 50 μm.

3D vertical assay [38] was performed starting with 2 × 10^5^ cells/ml resuspended in RPMI-1640 with 2.2 mg/ml type-I collagen, at pH 7.4, and seeded in 50 μl-aliquots in wells pre-coated with 3% heat-inactivated BSA. Cells were stained with YoPro and photographed along Z-axes through high-density collagen-I. Z-stack images were captured each 5 μm from the well bottom, up to 120 μm upward, toward the chemoattractant.

### Image acquisition and processing

Images for analysis of IF and F-actin staining were acquired in z-stack with NIS-Elements software (Nikon) and deconvoluted with the associated deconvolution software package, using a fluorescence microscope Nikon Eclipse Ti (Nikon) equipped with 40x DIC M Plan Fluor objective (Nikon) and DS-Qi1Mc-U2 12 bit (Nikon) camera.

Images of ICC on agar-embedded cells and of IHC: Olympus BX43 equipped with camera Olympus DV20 and Cell Sens Dimension software.

Chemotaxis images: Zeiss Axioskop 40 microscope (Zeiss) equipped with 5x objective (Zeiss), through AxioCam MRc 12 bit camera (Zeiss) and AxioVision software (Zeiss).

Chemokinesis live imaging: Nikon Eclipse Ti microscope (Nikon) and ORCA camera (Hamamatsu) equipped with 20x Ph2 DM Plan Apo objective (Nikon) and NIS-Elements software (Nikon). Images were analyzed with Manual Tracking plugin of ImageJ suite (NIH, USA), following each individual cell to the end of the experiment or to mitosis. Data were analyzed with Chemotaxis and Migration Tool 2.0 (Ibidi).

3D matrix invasion assays: acquired with NIS-Elements software (Nikon) and deconvoluted with the associated deconvolution software, using a Nikon Eclipse Ti microscope (Nikon). It was equipped with 4x Achromat objective (Nikon) and DS-Qi1Mc-U2 12 bit camera (Nikon) in case of spheroid assay; it was equipped with 20x Ph2 DM Plan Apo objective (Nikon) and ORCA camera (Hamamatsu) in case of vertical assay. Image analysis was performed with ImageJ software (NIH, USA).

### Statistical analysis

Data of protein IHC are indicated as mean ± SEM and analyzed with Student's *t* test for paired samples.

Data from gene expression analysis in cell lines and cell circularity, viability, adhesion, chemotaxis, chemokinesis and 3D matrix invasion assays were obtained from more than three independent experiments (each performed with triplicates). They are expressed as mean ± SEM and analyzed with one-way Anova test and Tukey's Multiple Comparison post-hoc test.

Gene expression analysis in patients’ samples (*N* versus *T*) was evaluated with the non-parametric Mann-Whitney test.

Protein expression correlation was analyzed with Pearson Correlation test, *r* = −0.6641.

Always, difference between means was judged statistically significant for *p* ≤ 0.05.

All statistical procedures were performed using GraphPad software.

## SUPPLEMENTARY MATERIALS FIGURES AND MOVIES






