# Supplementary material for: MICAL2 is a novel human cancer gene controlling mesenchymal to epithelial transition involved in cancer growth and invasion
Source: Oncotarget. 2015 Dec 12;7(2):1808–25. doi: 10.18632/oncotarget.6577 (PMC4811499; doi:10.18632/oncotarget.6577)
Supplement: Supplementary file 1 [file oncotarget-07-1808-s001.pdf]

MICAL2 is a novel human cancer gene controlling mesenchymal to epithelial transition involved in cancer growth and invasion

Supplementary Materials

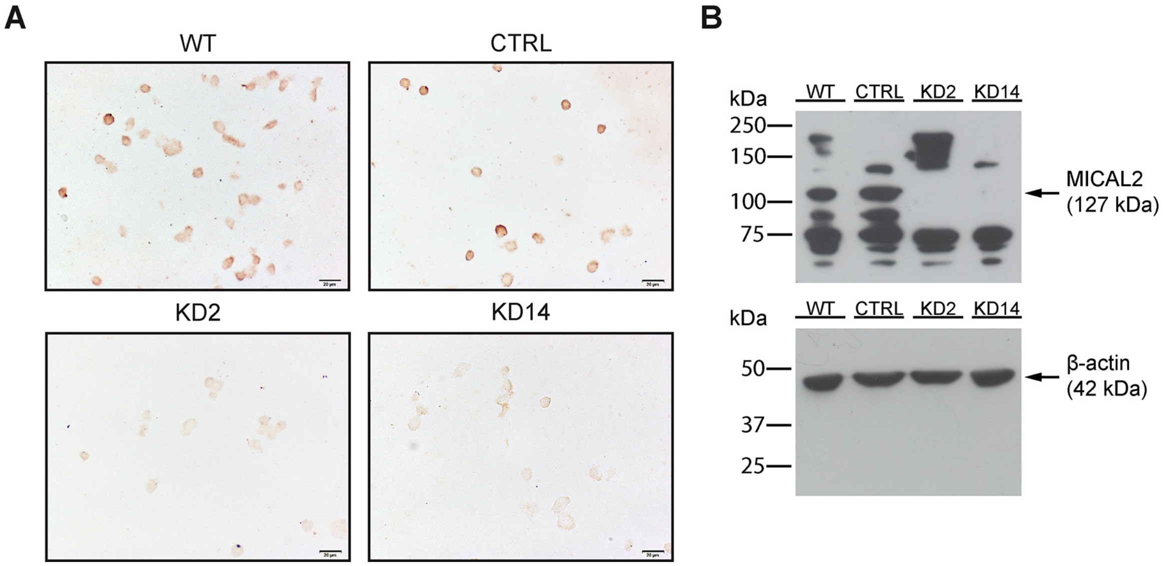

Supplementary Figure S1: Validation of rabbit anti-MICAL2 polyclonal antibody. Anti-MICAL2 antiserum was generated in our laboratory, as in [44], in HY/CR New Zealand White male rabbits (Charles River Laboratories Italia, Italy) immunized with the protein carrier Mariculture Keyhole Limpet Hemocyanin (mcKLH) conjugated with peptide NH_2-GTWQEQEAPRRDVPTESSC-COOH. The epitope, conserved in human and mouse, is highly specific for MICAL2, and located in a region of high diversity among the three MICAL homologs. Whole antiserum was used without affinity purification, at the dilutions indicated in the Materials and Methods section. Specificity was tested in several ways. (**A**) ICC with DAB staining on formalin-fixed, agar-embedded 786-O WT, MIC2-KD and CTRL cells (generated as described in legend to Supplementary Figure 4). A strong signal is obtained from WT and CTRL cells. The signal is barely detectable in MIC2-KD cells (KD2 and KD14 clonal populations). Ematossilin-Eosin staining was performed as quality control (not shown). (**B**) WB with 100 μg of whole cell lysates of 786-O WT, MIC2-KD and CTRL cells was performed to investigate the molecular weight of the antigen. Noticeably, MIC2-KD cells show lack of the 127 KDa expected band, that is present in turn in the WT and CTRL lanes (arrow). Other bands are present on the blot as expected from whole polyclonal rabbit antiserum. Loading control was performed with anti-beta actin antibody.

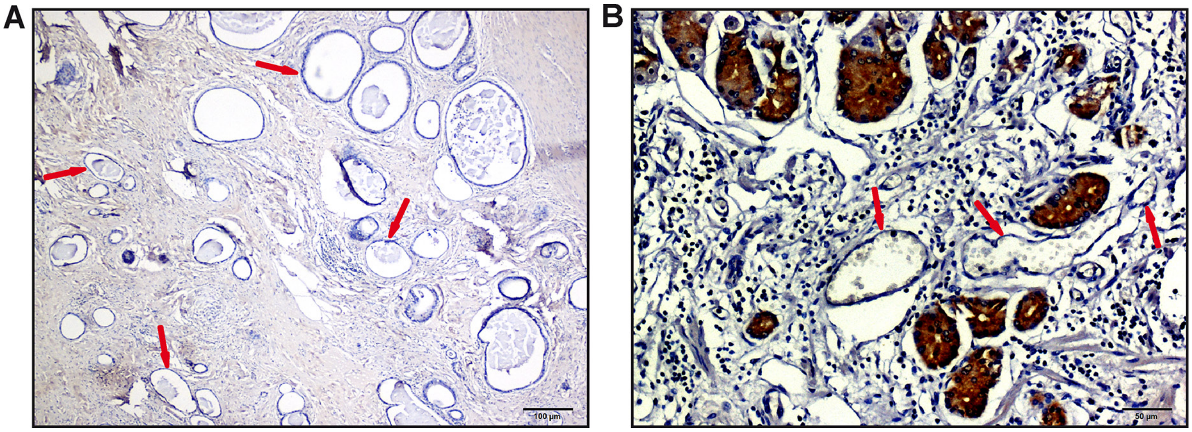

Supplementary Figure S2: Proteins of MICAL family display a different expression pattern in GC. IHC with DAB staining of histological section of human GC with (**A**) anti-MICAL1 (ECs were not positive, arrows. Cancer cells were completely negative), and (**B**) anti-MICAL3 (cancer cells were completely negative. ECs were negative as well, arrows). A strong signal derived from normal gastric glands.

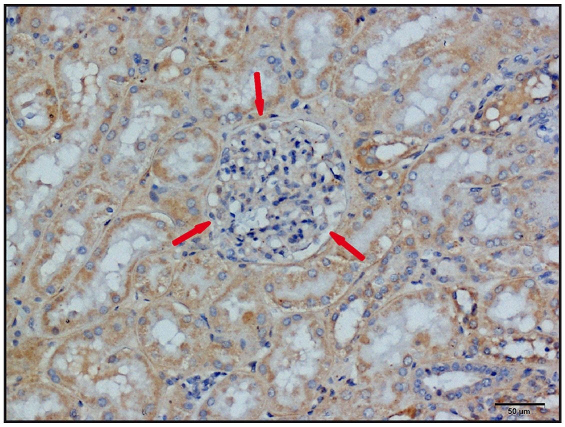

Supplementary Figure S3: Expression of MICAL2 protein in normal kidney glomeruli. MICAL2 was not detected in normal kidney glomeruli; a low expression was found in normal tubules (Scale bar: 50 μm).

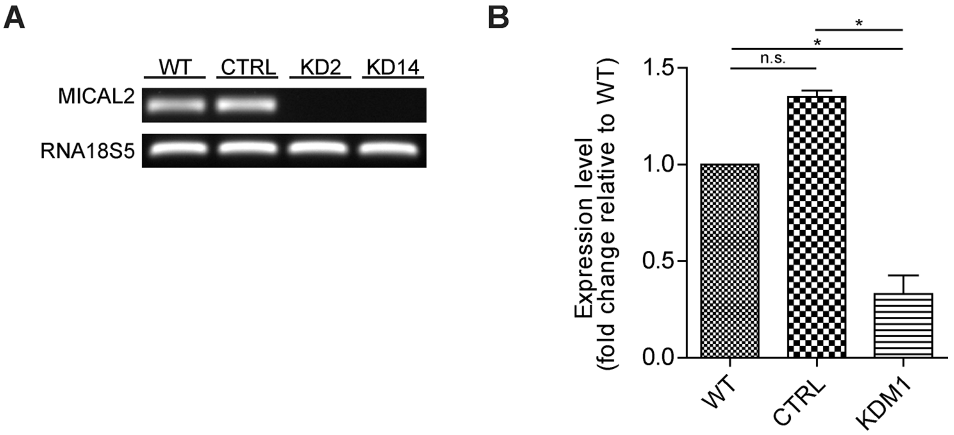

Supplementary Figure S4: Silencing MICAL2 in human cancer cell lines: generation and validation. The following constructs were used to abate MICAL2 *in vitro*: MISSION plasmids (Sigma-Aldrich, pLKO.1-Neo-CMV-tGFP + shRNA) expressing shRNA for human MICAL2 (TRCN0000046579, TRCN0000046580, TRCN0000046581). A MISSION plasmid expressing shRNA against murine MICAL2 (TRCN0000192634, Sigma-Aldrich) was used as negative control in 786-O and MDA-MB-231; the corresponding empty plasmid (pLKO.1-Neo-CMV-tGFP, Sigma-Aldrich) was used in MERO-14 cells. Plasmids were used alternatively circular or linearized with ScaI restriction enzyme (Invitrogen) that cuts a unique site in the gene for Ampycillin resistance. Cells were transfected with Lipofectamine2000 (Invitrogen), following the manufacturer's instructions. 48 hrs after the transfection, positive cells were selected with 500 µg/ml (786-O and MDA-MB-231) or 700 µg/ml (MERO-14) G418 (Sigma-Aldrich). From different transfection rounds, either pools or single clones of shRNA-expressing cells were obtained. Validation was performed at mRNA (QRT-PCR, see below), and protein (WB, ICC) level to show that knockdown at protein level was achieved (please see Supplementary Figure 1). A, In 786-O, highly effective abatement made the mRNA undetectable even after 44 cycles of QRT-PCR. Here a gel run is shown to visualize the negative result. MIC2-KD (clones KD2 and KD14) lanes do not show the expected RT-PCR product (215 bp). Loading control was performed with RNA18S5. B, In MERO-14, an abatement of 75% was obtained with respect to parental cells. The difference between WT and CTRL cells was not significant, while it was between reference cells and KDM1 (One-way Anova test and Tukey's Multiple Comparison post-hoc test. Horizontal lines denote mean and SEM. Ns: non significant. *p ≤ 0, 05, **p ≤ 0, 01, ***p ≤ 0, 001. The internal reference gene was RNA18S5).

Supplementary Movies 1 (786-O CTRL cells) and 2 (KD14 cells). MIC2-KD cells did not adopt a polarized arrangement in 2D assay. Directional cell migration requires cell polarity, with proper leading and trailing edges for the cell to explore the environment, make contact and detach to move forward. 786-O WT and CTRL cells migrated by extending dynamic protrusions, with polarized and persistent lamellipodia, retracted only to change direction of migration. They also showed well-defined trailing and leading edges, formed and retracted several times during migration (Movie 1). In contrast, most MIC2-KD cells showed large, highly dynamic but irregularly shaped lamellipodia, often broader than lamellipodia of control cells, that sometimes spread around most of the cell periphery, with quasi-radial symmetry and without formation of visible trailing edge. Multiple, narrow and small protrusions elongated from the lamellipodium formed by MIC2-KD cells, often lacking an elongated trailing edge and showing round cell shape (Movie 2). The net result was that cells did not move directionally, as if 'sliding' on the adhesion surface. Other times, they were surrounded by whole radial lamella without projection of effective leading or trailing edges, and without oriented movement.
